# Biofortified Maize Improves Selenium Status of Women and Children in a Rural Community in Malawi: Results of the Addressing Hidden Hunger With Agronomy Randomized Controlled Trial

**DOI:** 10.3389/fnut.2021.788096

**Published:** 2022-01-06

**Authors:** Edward J. M. Joy, Alexander A. Kalimbira, Joanna Sturgess, Leonard Banda, Gabriella Chiutsi-Phiri, Hastings Manase, Jellita Gondwe, Elaine L. Ferguson, Zione Kalumikiza, Elizabeth H. Bailey, Scott D. Young, Limbanazo Matandika, Joseph Mfutso-Bengo, Kate Millar, Maja Niksic, Lucia Segovia de la Revilla, Blessings H. Likoswe, John C. Phuka, Felix P. Phiri, R. Murray Lark, Dawd Gashu, Simon C. Langley-Evans, E. Louise Ander, Nicola M. Lowe, Alan D. Dangour, Patson C. Nalivata, Martin R. Broadley, Elizabeth Allen

**Affiliations:** ^1^Faculty of Epidemiology and Population Health, London School of Hygiene & Tropical Medicine, London, United Kingdom; ^2^Bunda College, Lilongwe University of Agriculture and Natural Resources, Lilongwe, Malawi; ^3^Natural Resources College, Lilongwe University of Agriculture and Natural Resources, Lilongwe, Malawi; ^4^National Nutrition Reference Lab, Community Health Sciences Unit, Public Health Institute of Malawi, Lilongwe, Malawi; ^5^School of Biosciences, University of Nottingham, Sutton Bonington Campus, Loughborough, United Kingdom; ^6^School of Public Health and Family Medicine, College of Medicine, University of Malawi, Blantyre, Malawi; ^7^Department of Nutrition, HIV and AIDS, Ministry of Health, Lilongwe, Malawi; ^8^Center for Food Science and Nutrition, Addis Ababa University, Addis Ababa, Ethiopia; ^9^Inorganic Geochemistry, Centre for Environmental Geochemistry, British Geological Survey, Nottingham, United Kingdom; ^10^UCLan Research Centre for Global Development, University of Central Lancashire, Preston, United Kingdom; ^11^Rothamsted Research, Harpenden, United Kingdom

**Keywords:** selenium, fertilizer, nutrition, agriculture, biofortification

## Abstract

**Background:** Selenium deficiency is widespread in the Malawi population. The selenium concentration in maize, the staple food crop of Malawi, can be increased by applying selenium-enriched fertilizers. It is unknown whether this strategy, called agronomic biofortification, is effective at alleviating selenium deficiency.

**Objectives:** The aim of the Addressing Hidden Hunger with Agronomy (AHHA) trial was to determine whether consumption of maize flour, agronomically-biofortified with selenium, affected the serum selenium concentrations of women, and children in a rural community setting.

**Design:** An individually-randomized, double-blind placebo-controlled trial was conducted in rural Malawi. Participants were randomly allocated in a 1:1 ratio to receive either intervention maize flour biofortified with selenium through application of selenium fertilizer, or control maize flour not biofortified with selenium. Participant households received enough flour to meet the typical consumption of all household members (330 g *capita*^−1^ day^−1^) for a period of 8 weeks. Baseline and endline serum selenium concentration (the primary outcome) was measured by inductively coupled plasma mass spectrometry (ICP-MS).

**Results:** One woman of reproductive age (WRA) and one school-aged child (SAC) from each of 180 households were recruited and households were randomized to each group. The baseline demographic and socioeconomic status of participants were well-balanced between arms. No serious adverse events were reported. In the intervention arm, mean (standard deviation) serum selenium concentration increased over the intervention period from 57.6 (17.0) μg L^−1^ (*n* = 88) to 107.9 (16.4) μg L^−1^ (*n* = 88) among WRA and from 46.4 (14.8) μg L^−1^ (*n* = 86) to 97.1 (16.0) μg L^−1^ (*n* = 88) among SAC. There was no evidence of change in serum selenium concentration in the control groups.

**Conclusion:** Consumption of maize flour biofortified through application of selenium-enriched fertilizer increased selenium status in this community providing strong proof of principle that agronomic biofortification could be an effective approach to address selenium deficiency in Malawi and similar settings.

**Clinical Trial Registration:** http://www.isrctn.com/ISRCTN85899451, identifier: ISRCTN85899451.

## Introduction

Selenium (Se) deficiency is widespread in Malawi. In a recent national survey (1), 62.5% of women of reproductive age (WRA; *n* = 802) had plasma Se concentrations below a threshold for the optimal activity of the Se-containing protein glutathione peroxidase 3 (GPX3; <84.9 μg L^−1^) which plays a role in antioxidant function. Selenium is important for health and immunity, and deficiency may lead to sub-optimal thyroid function, impaired cognitive development in children, increased risk of anemia, and increased risk of stroke (2–9).

Across most of Malawi, the concentration of Se in the staple crop maize grain is small (median 0.0188 mg kg^−1^, *n* = 1,806) (10) and this underlies inadequate dietary Se intakes leading to the widespread Se deficiency that is observed directly in blood and urine biomarkers (1, 7, 10–16). Low concentrations of Se in maize and other locally-grown crops results from their production on agricultural soils which are typically highly weathered and acidic with low concentrations of plant-available Se (10, 13, 14). There are, however, some areas of Malawi where the risk of Se deficiency is small, notably areas close to Lake Malawi and parts of southern Malawi, and this is likely due to the surrounding soil types with large concentrations of plant-available Se resulting in naturally high concentrations in maize grain (c. 0.3 mg kg^−1^), or potentially greater consumption of fish which is a good source of multiple micronutrients including Se (1, 11–13).

There are various potential strategies to alleviate Se deficiency including: the promotion of increased consumption of animal-source foods, fortification of foods at processing stage and specific nutrient supplementation programmes (17). An alternative approach—known as agronomic biofortification—is to increase the Se concentration in staple food crops using Se-enriched fertilizers in areas where there is inherently low soil-to-crop Se transfer (18). This approach might be particularly valuable in rural sub-Saharan Africa because it could reach subsistence households whose low purchasing power constrain their access to animal-source foods or foods fortified at processing stage. Application via granular fertilizers or foliar spray of relatively small amounts of selenate-Se (at a rate of c. 5 g Se ha^−1^) has been shown to increase Se concentration in maize grain from 0.061 to 0.156 mg kg^−1^ (19).

Studies testing the effect of Se biofortified staple crops on micronutrient status provide limited evidence to-date (20). Alfthan et al. (21) report observational (albeit compelling) data from Finland showing an association of Se fertilizer policy with population Se levels, Wu et al. (22) tested biscuits made with Se-biofortified wheat flour among Australian adult males, and Hurst et al. (23) tested Se-biofortified onions among UK adults aged 50–64 years. There is currently no evidence from studies testing whether the consumption of agronomically biofortified maize affects the Se status of women and children in rural sub-Saharan Africa where deficiency is endemic. Governments require this information if they are to identify effective pathways to resolve nutrient insufficiency.

The Addressing Hidden Hunger with Agronomy trial (AHHA) tested the effect of consumption of maize flour agronomically-biofortified with Se on the Se concentration in blood serum of women and children in a community setting in Malawi. This trial was conducted among adult women of reproductive age (WRA) and primary school-age children (SAC) in an area of rural Malawi with prevalent Se deficiency.

## Materials and Methods

The study protocol was reported previously (24), and a brief description is provided here. The study is reported according to CONSORT guidelines (25) (Supplementary Table 1).

### Study Setting

The trial was conducted in Traditional Authority (TA) Wimbe, Kasungu District, in central Malawi. The study area was selected because of the high prevalence of Se deficiency, with >90% probability of deficiency against the threshold for optimal GPX3 expression (1). Most households in the area rely on subsistence farming and tobacco production. Formative research, conducted a year before trial recruitment, informed the AHHA trial design and delivery (26).

### Study Design and Trial Participants

The AHHA trial was an individually randomized, parallel group, two-arm, double-blind, placebo controlled trial, with participants allocated to treatment arms in a 1:1 ratio (24). The trial was powered to detect an increase in Se concentration in serum of 4.9 μg L^−1^ with 5% significance and 90% power, allowing for 20% dropout, based on a standard deviation of 9.0 μg L^−1^ (standardized difference of 0.54). This gave a target sample size of 90 households (each with one WRA and one SAC) per trial arm.

As reported in the study protocol (24), the trial was originally designed as a three-arm trial, whereby the third arm would receive Zn-biofortified maize flour. However, the response of the maize grain Zn concentration to the application of Zn fertilizer was lower than anticipated and the objectives were revised to focus on the Se arm only. The protocol was amended and approved by the relevant research ethics committees.

### Eligibility

In May 2019 trained Research Assistants (RAs) recruited by Lilongwe University of Agriculture and Natural Resources (LUANAR) conducted a census to create a list of all households in the study area (*n* = 1,179) and a roster of all household members.

Eligible households were those with at least one non-pregnant (self-reported) WRA (age 20–45 years) and at least one SAC (age 5–10 years) in residence during July to October 2019, with meals typically prepared and consumed at home, and where the household-head agreed that the household would receive and consume maize flour for the duration of the intervention.

To be eligible, WRA had to be willing and able to provide consent, and the parent or guardian of the participant SAC had to be willing and able to provide consent on their behalf.

### Household Selection and Randomisation

All 1,179 households in the census were assessed and 553 households were identified as potentially eligible. The statistician (EA), based remotely in the Clinical Trials Unit (CTU) at LSHTM, randomly selected 180 households, from those identified as eligible, that were then fully informed about the trial and invited to participate. Recruitment occurred between 24th June 2019 and 6th July 2019. One participant WRA and one participant SAC from each household was included in the trial even though flour was provided to the whole household. During recruitment, it became apparent that some households listed as eligible during the census were in fact ineligible. This was mainly due to households misreporting the presence of children in the eligible age range. A further 69 households were randomly selected from the original list to replace the households found to be ineligible. Where households had multiple eligible WRA and/or SAC, the RAs wrote the names on small pieces of paper, scrunched these up and mixed in a bowl, then drew one WRA and one SAC at random. Permission of household heads and individual consent of participants were obtained by teams of trained RAs prior to recruitment.

Following baseline data and sample collection the statistician used a computer-based randomization programme to randomly allocate households in a 1:1 ratio to receive either Se agronomically-biofortified flour (*n* = 90) or control flour (*n* = 90).

Intervention allocation was concealed from participants, RAs conducting surveys, laboratory analysts and those involved in managing or overseeing the trial. Only the supervisor of the maize flour distribution and a data manager in the CTU at LSHTM knew the allocation of households to treatments. The treatment allocations were unblinded after statistical analyses were complete.

### Intervention

Control and intervention maize was grown for this study by the research team at LUANAR, Bunda Campus, Malawi, with Se fertilizer applied via the soil (24). Grain was milled at LUANAR to produce degermed and dehulled maize flour commonly called *Granmil* flour. Samples of control and intervention flour were taken from three sacks after milling and analyzed by ICP-MS, finding mean Se concentrations of 0.010 and 0.170 mg kg^−1^, respectively. The concentrations of other elements measured by ICP-MS did not differ between the control and intervention flour. The Se concentration in intervention maize was similar to concentrations measured in maize grain grown conventionally on farmers' fields on Eutric Vertisol soils in Malawi (13). Eutric Vertisols are commonly found in parts of the Shire Valley and along the shore of Lake Malawi, where population Se levels are typically adequate. Selenium is readily available for uptake by plants in these soils because of their characteristic properties including their pH (>6.5).

Household maize flour requirements were calculated on the basis of estimated consumption of 330 g *capita*^−1^ day^−1^ for all household members over the age of 1 year (equivalent to 10 kg *capita*^−1^ month^−1^). The estimate of requirement was derived from Food Balance Sheets (27), which report 342 g *capita*^−1^ day^−1^ of “Maize and products” available for consumption, and national household survey data (11), which report mean consumption of 347 g Adult Male Equivalent^−1^ day^−1^ of ‘Maize flour refined'. Thus, consumption of 330 g of intervention flour would provide ~60 μg *capita*^−1^ day^−1^ of Se, while the same quantity of control flour would provide <5 μg *capita*^−1^ day^−1^ of Se. This compares with an Estimated Average Requirement (EAR) of 45 μg day^−1^ for adult women, and 23 μg day^−1^ for children age 4–8 years (28). The EAR is the quantity of a nutrient that is estimated to meet the requirement of half the healthy individuals in an age- and sex-specific group (29).

Flour was pre-packaged in 10, 5, and 2.5 kg bags and households were given the appropriate number of bags of different sizes to meet their need (rounded up to the nearest 2.5 kg). The original intention was to provide 12 weeks of maize flour, with the end-line survey conducted 10 weeks after initiation of treatment (24). However, we underestimated the loss of mass due to discarding the bran at grain milling—we estimated this would be ~25% whereas it was actually 41%—which meant insufficient agronomically-biofortified flour was available for a 12-week intervention. The intervention was, therefore, shortened to 8 weeks with households participating in the endline survey after households participating in the endline survey 6.8 weeks (mean 47.3 ± 0.2 days) after receiving their first distribution of flour. All participant households received non-biofortified flour for an additional 4 weeks after the intervention period, because at recruitment they were informed they would receive flour for 12 weeks.

The trial statistician (EA) generated a set of unique seven-digit codes that were used to label each bag of study flour. These unique identification codes (UIDs) included an embedded indicator for the contents i.e., either intervention or control flour. Only the supervisor of the flour distribution team and a data manager based in the CTU at LSHTM had access to the code structure. This allowed for correct allocation and subsequent checking of flour allocation at distribution.

Flour was distributed at pre-agreed centralized distribution points at 2-weekly intervals beginning ~2 weeks after recruitment and the baseline survey (18th July 2019). Participants received help transporting maize flour to their home if necessary. Flour bag UIDs and household codes were recorded at flour distribution, and these were checked against an allocation list by an independent data manager at LSHTM. Errors of allocation were reported directly to the supervisor of the flour distribution team to enable them to correct their procedures in subsequent flour distributions.

Non-participating households in the study area also received non-biofortified flour for the duration of the study. This “control-equivalent” flour was not labeled with codes and was distributed separately. The decision to provide flour to non-participant households was based on findings from formative research (26), which suggested that providing flour to all households in the area would be considered “fair” by the community. It would reduce the likelihood of sharing flour between household and it would avoid risk of envy or stigma.

### Outcomes

#### Primary Outcome

- Se concentration in blood serum (μg L^−1^).

#### Secondary Outcomes

- Prevalence of Se deficiency, defined using established thresholds of deficiency (i.e., <84.9 μg L^−1^, <64.8 μg L^−1^ and <30 μg L^−1^) (30)
- Concentration in serum of the markers of inflammation alpha (1)-acid glycoprotein (AGP) (mg L^−1^) and C-reactive protein (CRP) (mg L^−1^)
- Concentration of hemoglobin in whole venous blood (g dL^−1^)
- Prevalence of anemia, defined using standard thresholds (31)
- Dietary Se intakes among WRA (μg day^−1^) and the % at risk of inadequate intakes
- Self-reported morbidity outcomes: Diarrhoea incidence, severity and duration, incidence of vomiting and incidence of fever for WRA and SAC, and incidence of pneumonia among SAC.

### Data Collection Procedures

Baseline and endline demographic and dietary data were collected in participants' households. Participants were then directed to a nearby mobile field clinic where anthropometric measurements were taken by RAs from LUANAR and blood samples collected and processed by nursing and laboratory staff from the Community Health Sciences Unit, Lilongwe, Malawi (CHSU). Dietary data (24-h recalls) were collected from WRA in their homes during baseline and endline surveys, with repeated recalls collected from 10% of randomly selected participants. The percentage of WRA at risk of inadequate Se intakes was estimated using the software Intake Modeling, Assessment, and Planning Program (IMAPP), which estimates the percentage at risk of inadequate intakes and above the upper tolerable level (UL) using the fixed cut-point approach after adjusting the nutrient intake distribution to simulate a usual intake distribution. The harmonized EAR for WRA were used (i.e., 45 and 59 μg day^−1^ for lactating women) and the UL was 400 μg day^−1^.

All participants were assigned a unique numeric ID that was used in data capture forms and subsequent analyses to maintain anonymity. Questionnaire and anthropometry data were collected on passcode-protected tablets using an Open Data Kit (ODK). Participant IDs were matched to sample tube codes via ODK. Completed forms were uploaded to a secure cloud server daily and were encrypted for security. The trial manager (LB) and data manager (JS) reconciled samples and data forms daily.

Whole venous blood was drawn into trace element–free tubes (BD Diagnostics, Eysins, Switzerland); after clotting, the serum was separated by centrifugation within 40 min. Serum 0.5 mL aliquots were transferred into cryovials, and transported in a cold chain to CHSU where they were stored at −80°C. Serum samples were subsequently shipped on dry ice to the University of Nottingham, UK, under a Material Transfer Agreement. Baseline and endline samples were analyzed at the same time.

### Analysis of Blood Serum for Se

The concentration of Se was measured by ICP-MS (Thermo Fisher Scientific iCAPQ, Thermo Fisher Scientific, Bremen, Germany) at the University of Nottingham using methods specified in Belay et al. (32). In brief, samples were diluted 1 in 31 in 0.5% HNO_3_ (Primar Plus™ grade). The ICP-MS was operated in “collision-reaction cell mode,” with kinetic energy discrimination, using H_2_ as the cell gas to maximize the sensitivity for Se determination. Calibration was undertaken using a multi-element standard (Claritas-PPT grade CLMS-2; SPEX Certiprep Inc., Metuchen, NJ, USA). Correction for drift was undertaken using three internal standards (^72^Ge, ^103^Rh, ^185^Re in 0.5% HNO_3_, and 4% methanol). The limit of detection (LOD) for Se was 0.034 ng mL^−1^, representing 3× the standard deviation (SD) of 10 operational blanks. Accuracy was verified using appropriate reference materials (Seronorm-L1 and Seronorm-L2; Nycomed Pharma AS, Billingstad, Norway). Mean recoveries were 96.5% (SD = 2.3%) and 96.9% (SD = 2.9%) for Seronorm-L1 (Lot 1309438; accredited value 86.5 ng mL^−1^) and Seronorm-L2 (Lot 1309416; accredited value 137 ng mL^−1^), respectively (*n* = 47).

### Monitoring

Adherence to the intervention and experience of any adverse events were recorded by trained RAs four times through household visits during the intervention and at endline. To measure adherence, a household member responsible for meal preparation was asked whether the allocated trial flour was used the previous day for each meal and whether/what quantity of non-study flour was used by the household over the past 2 weeks. Participant WRA and the parents or guardians of SAC were asked to report experience of any adverse events including fever, diarrhoea, and coughing and these were recorded by RAs on an electronic form. Participants were able to report serious adverse events to the study coordination centre (LUANAR) or to Research Assistants and volunteer health extension workers (24).

### Statistical Analysis

The primary analysis was by intention-to-treat with each participant analyzed according to the treatment arm they were allocated to irrespective of the treatment received. Each outcome measure was analyzed using separate Generalized Linear Models (GLM). Statistical significance for these analyses was taken at the 5% level (*p* < 0.05; two-tailed). Analyses were carried out adjusted for baseline status. Further adjustment for inflammation status was not made due to missing CRP and AGP concentration data (see results section). The statistical analysis plan was approved by the Trial Steering Committee that included an independent statistician.

For serum Se concentration (the primary outcome) and hemoglobin concentration, analysis of covariance (adjusting for baseline values) was used to estimate a mean difference between the two arms of the trial, with non-parametric bootstrapping used to allow for any non-normality of the outcome and to estimate bias corrected 95% confidence intervals.

For binary outcomes, generalized linear models were used with a log link to examine the effect of the intervention. For continuous outcomes, we report unadjusted and adjusted mean differences with 95% confidence intervals (CI). For binary outcomes, we report unadjusted and adjusted relative risk with 95% CI.

We conducted pre-specified subgroup analyses by (i) 10-year age groups, (ii) lactation status, and (iii) pregnancy status (note some WRA became pregnant between recruitment and endline). Evidence for any differential effects of the intervention on the primary and secondary outcomes by these subgroups was assessed by a “treatment by subgroup” interaction term. Where there was evidence of an interaction the effects in the different subgroups were estimated directly from the regression model with the interaction term included.

### Ethical Approval

Ethical approval for the study (including protocol amendments) was granted by the University of Malawi's College of Medicine Research Ethics Committee (reference P.11/18/2539) and the London School of Hygiene & Tropical Medicine Interventions Research Ethics Committee (reference 16181). The study was conducted in accordance with the ethical standards set out in the Declaration of Helsinki (33). All participants provided written informed consent, with the parents of guardians of SAC providing consent on their behalf.

## Results

**Figure 1:**
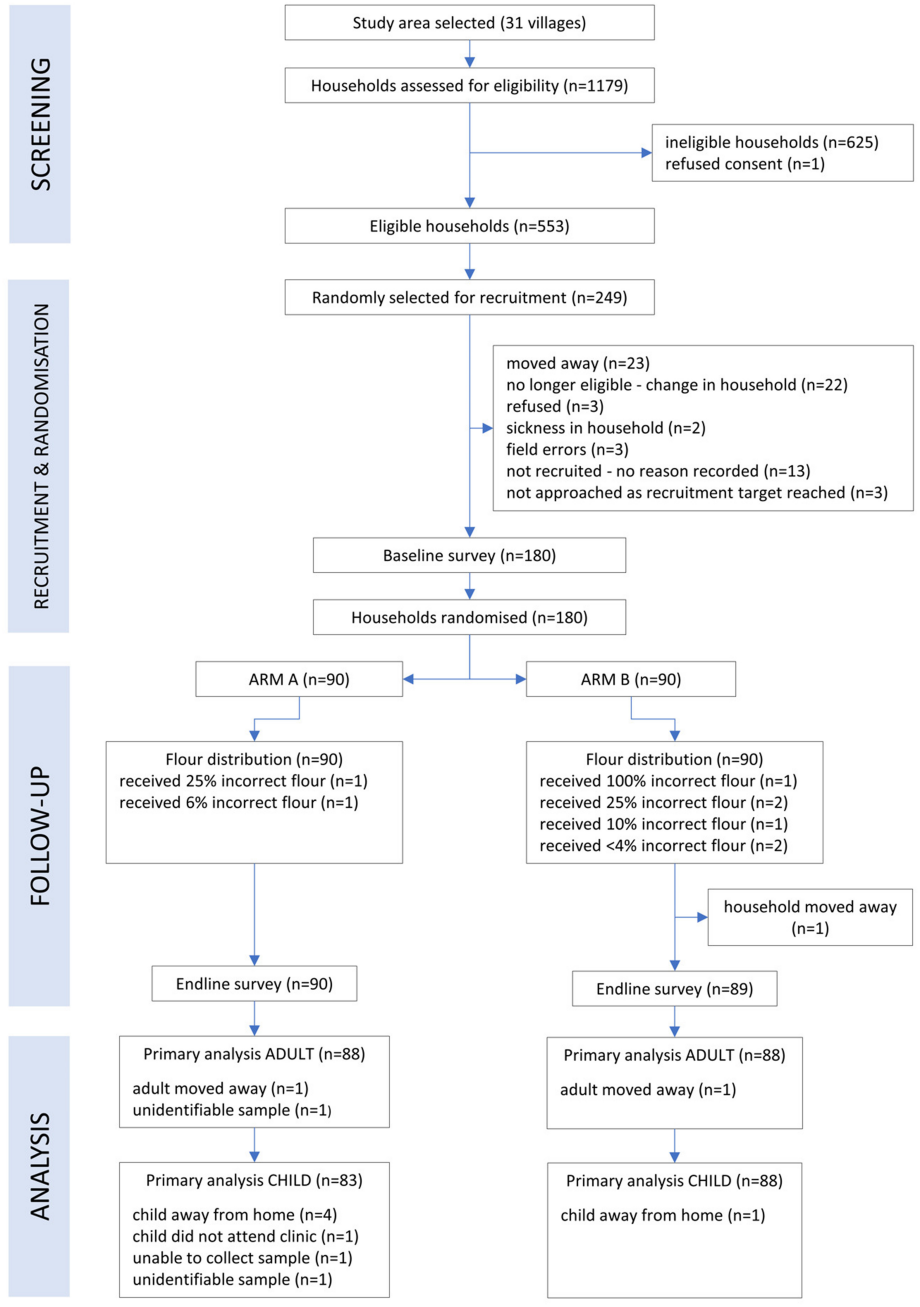
CONSORT flow diagram of participants in the AHHA trial.

Following recruitment and the baseline survey, participants were randomly allocated to treatment arms on 8th July 2019. A total of 1,179 households were assessed for eligibility, 553 were eligible and 180 were randomized into the trial, each containing one WRA and one SAC. At endline we had complete data on 176 WRA and 171 SAC (Figure 1).

Participant age, weight and height were well-balanced between arms (Table 1). Among WRA, the prevalence of severe and moderate anemia was low (8%) and no SAC had severe anemia. At baseline, serum Se status was balanced between trial arms among both WRA and SAC (Table 2).

**Table 1:** Baseline characteristics of households, women of reproductive age (WRA) and school-aged children (SAC) participating in the AHHA trial.

	**Control**	**Intervention**
	***n* = 90**	***n* = 90**
**Households**		
Household size, mean (SD), range	6.6 (1.8), 3–13	6.6 (1.9), 3–14
Adults	2.8 (1.1), 1–6	3.0 (1.3), 1–8
Children >1 year	3.8 (1.4), 1–7	3.6 (1.3), 1–6
**Women of reproductive age (WRA)**		
Age (years), mean (SD)	32.0 (7.1)	34.8 (6.8)
Height (cm), mean (SD)	156.2 (6.0)	155.4 (6.5)
	1 missing value	
Weight (kg), mean (SD)	57.0 (8.8)	56.8 (9.6)
	1 missing value	
**Anemia status**, ***n*** **(%)**		
Non-anemic^a^	60 (68.2)	61 (68.3)
Mild^c^	22 (25.0)	19 (21.6)
Moderate^e^	6 (6.8)	7 (8.0)
Severe^f^	0	1 (1.1)
	2 missing values	2 missing values
**School age child (SAC)**		
Age (years), mean (SD)	7.6 (1.7)	7.5 (1.7)
Height (cm), mean (SD)	117.8 (9.2)	118.5 (9.7)
	1 missing value	
Weight (kg), mean (SD)	21.9 (3.8)	22.5 (4.5)
	1 missing value	
**Anemia status**, ***n*** **(%)**		
Non-anemic^b^	48 (55.2)	51 (58.6)
Mild^d^	8 (9.2)	13 (14.9)
Moderate^e^	31 (35.6)	23 (26.4)
Severe^f^	0	0
	3 missing values	3 missing values

a *Serum hemoglobin concentration ≥12.0 g dL^−1^.*

b *Serum hemoglobin concentration ≥11.5 g dL^−1^.*

c *Serum hemoglobin concentration 11.0–11.9 g dL^−1^.*

d *Serum hemoglobin concentration 11.0–11.4 g dL^−1^.*

e *Serum hemoglobin concentration 8.0–10.9 g dL^−1^.*

f *Serum hemoglobin concentration <8.0 g dL^−1^*.

**Table 2:** Mean (standard deviation) serum selenium concentration at baseline and endline and the mean difference (bias corrected 95% confidence interval) at endline between trial arms, among women of reproductive age (WRA) and school-aged children (SAC) participating in the AHHA trial.

	**Control**		**Intervention**		
	**Baseline serum selenium concentration (μg L^−1^)**	**Endline serum selenium concentration (μg L^−1^)**	**Baseline serum selenium concentration (μg L^−1^)**	**Endline serum selenium concentration (μg L^−1^)**	**Difference in serum selenium concentration (μg L^−1^)**
WRA	61.0 (23.1)	61.0 (13.5)	57.6 (17.0)	107.9 (16.4)	49.0 (45.9, 52.1)
	*n* = 89	*n* = 88	*n* = 88	*n* = 88	*n* = 173
SAC	50.4 (18.1)	50.8 (12.5)	46.4 (14.8)	97.1 (16.0)	48.7 (45.0, 51.8)
	*n* = 87	*n* = 83	*n* = 86	*n* = 88	*n* = 166

### Primary Outcome

After 8 weeks of intervention, we identified a statistically significant difference in serum Se concentrations between control and intervention arms for both WRA and SAC (Table 2). Serum Se concentration approximately doubled in the intervention arm between baseline and endline surveys in both WRA and SAC, while there was no change in the control arm in either demographic group. The mean difference in Se concentration between arms at endline, adjusted for baseline Se concentration, was 49.0 μg L^−1^ (95% CI: 45.9, 52.1) for WRA and 48.7 μg L^−1^ (95% CI: 45.0, 51.8) for SAC.

There was no evidence that the effect of the intervention differed among the pre-specified subgroups, 10-year age group (*p* = 0.245), pregnancy status (*p* = 0.278), or lactation status *p* = 0.249 (Supplementary Table 2).

### Secondary Outcomes

In line with the primary finding, the intervention was found to reduce markedly the prevalence of Se deficiency among both WRA and SAC (Table 3). Compared with the control arm, the relative risk of deficiency in the intervention arm was 0.07 (95% CI: 0.03, 0.15) and 0.20 (95% CI: 0.13, 0.30) among WRA and SAC, respectively, against a threshold of Se deficiency defined for the optimal activity of the antioxidant enzyme GPX3.

**Table 3:** Prevalence of selenium deficiency among women of reproductive age (WRA) and school-aged children (SAC) participating in the AHHA trial.

	**Control**		**Intervention**		
	**Baseline, *n* (%)**	**Endline, *n* (%)**	**Baseline, *n* (%)**	**Endline, *n* (%)**	**Relative risk (95% CI)**
**Prevalence of Se deficiency, GPX3**					
WRA	83/89 (93.3)	85/88 (96.6)	81/88 (92.1)	6/88 (6.8)	0.07 (0.03, 0.15)
SAC	84/87 (96.6)	81/83 (97.6)	86/86 (100)	17/88 (19.3)	0.20 (0.13, 0.30)
**Prevalence of Se deficiency, DIO**					
WRA	62/89 (69.7)	63/88 (71.8)	58/88 (65.9)	1/88 (1.1)	0.02 (0.002, 0.112)
SAC	77/87 (88.5)	77/83 (92.7)	77/86 (89.5)	2/88 (2.8)	0.02 (0.006, 0.097)
**Prevalence of Se deficiency, KD**					
WRA	3/89 (3.4)	0/88 (0)	4/88 (4.6)	0/88 (0)	NA
SAC	5/87 (5.8)	3/83 (3.6)	11/86 (12.8)	0/88 (0)	NA

*Deficiency is defined by thresholds of serum selenium concentration in adults for the optimal expression of glutathione peroxidase (GPX3; >84.9 mg L^−1^) and iodothyronine deiodinase (DIO; >64.8 mg L^−1^), and increased risk of Keshan Disease (KD, <30 mg L^−1^). Thresholds based on Thomson (30)*.

There was no evidence of an effect of the intervention on hemoglobin concentrations in either WRA or SAC [mean difference −0.02 (95% CI: −0.30, 0.20); −0.04 (95% CI: −0.30, 0.21)] (Table 4) and there was no evidence that the intervention reduced the risk of anemia among these populations (Table 5).

**Table 4:** Mean (standard deviation) hemoglobin (Hb) concentration in whole blood and the mean (bias corrected 95% confidence interval) difference at endline between trial arms, among women of reproductive age (WRA) and school-aged children (SAC) participating in the AHHA trial.

	**Control**		**Intervention**		
	**Baseline Hb concentration (g dL^−1^)**	**Endline Hb concentration (g dL^−1^)**	**Baseline Hb concentration (g dL^−1^)**	**Endline Hb concentration (g dL^−1^)**	**Difference in Hb concentration (g dL^−1^)**
WRA	12.7 (1.3)	12.9 (1.3)	12.5 (1.3)	12.6 (1.3)	−0.02 (−0.30, 0.20)
	*n* = 88	*n* = 88	*n* = 88	*n* = 88	*n* = 172
SAC	11.5 (1.4)	11.8 (1.3)	11.6 (1.2)	11.9 (1.2)	−0.04 (−0.30, 0.21)
	*n* = 87	*n* = 83	*n* = 87	*n* = 88	*n* = 167

**Table 5:** Prevalence of anemia among women of reproductive age (WRA) and school-aged children (SAC) participating in the AHHA trial.

	**Control**		**Intervention**		
	**Baseline, *n* (%)**	**Endline, *n* (%)**	**Baseline, *n* (%)**	**Endline, *n* (%)**	**Relative risk (95% CI)**
WRA	28/88 (31.8)	25/88 (28.4)	27/88 (30.7)	19/88 (21.6)	0.76 (0.45, 1.28)
					*n* = 176
SAC	39/87 (44.8)	35/83 (42.2)	36/87 (41.4)	27/88 (30.7)	0.73 (0.49, 1.09)
					*n* = 171

*Anemia is defined as hemoglobin concentration <12.0 g dL^−1^ among women of reproductive age (WRA) and <11.5 g dL^−1^ among school aged children (SAC) (19)*.

At endline, dietary Se intakes (mean ± SD) were greater among participant WRA in the intervention arm (85.4 ± 43.5 μg day^−1^) compared to those in the control arm (28.4 ± 28.5 μg day^−1^). The mean difference in dietary Se intake between arms at endline was 57.6 μg day^−1^ (95% CI: 46.4, 68.9) (Table 6). There was no evidence that the effect of the intervention differed between the pre-specified subgroups (Supplementary Table 3). At endline 83.8% of WRA were at risk of inadequate dietary Se intakes in the control arm and 10.2% in the intervention arm. At endline, for control and intervention groups, respectively, the median (25th, 75th) intake of Se from maize flour was 3.40 (2.80, 4.10) μg day^−1^ and 62.6 (47.6, 74.5) μg day^−1^, while the median (25th, 75th) contribution of maize flour to total dietary Se was 27.2% (9.1, 45.2%) and 85.6% (69.1, 92.8%).

**Table 6:** Mean (standard deviation) dietary selenium intake and the mean (bias corrected 95% confidence interval) difference at endline between trial arms, among women of reproductive age (WRA) participating in the AHHA trial.

	**Control**		**Intervention**		
	**Baseline intake (μg day^−1^)**	**Endline intake (μg day^−1^)**	**Baseline intake (μg day^−1^)**	**Endline intake (μg day^−1^)**	**Difference in intake (μg day^−1^)**
WRA	28.4 (28.5)	30.3 (35.5)	25.8 (26.3)	85.4 (43.5)	57.6 (46.4–68.9)
	*n* = 88	*n* = 88	*n* = 89	*n* = 89	*n* = 177

There was no evidence that the intervention reduced the risk of diarrhoea (including severe and persistent episodes), vomiting, or pneumonia (Table 7).

**Table 7:** Incidence of diarrhoea, vomiting, fever, and pneumonia at endline among women of reproductive age (WRA) and school-aged children (SAC) participating in the AHHA trial.

	**Control**		**Intervention**		
	**Baseline, *n* (%)**	**Endline, *n* (%)**	**Baseline, *n* (%)**	**Endline, *n* (%)**	**Relative risk (95% CI)**
**Prevalence of diarrhoea, total**					
WRA	2/90 (2.2)	3/90 (3.3)	5/90 (5.6)	8/89 (9.0)	2.70 (0.74, 9.84)
SAC	6/90 (6.7)	2/90 (2.2)	10/90 (11.1)	3/89 (3.4)	1.52 (0.26, 8.86)
**Prevalence of severe diarrhoea^a^**					
WRA	2/90 (2.2)	0/90 (0)	4/90 (4.4)	3/89 (3.4)	NA
SAC	2/90 (2.2)	0/90 (0)	1/90 (1.1)	0/89 (0)	NA
**Prevalence of persistent diarrhoea^b^**					
WRA	1/90 (1.1)	0/90 (0)	0/90 (0)	3/89 (3.4)	NA
SAC	1/90 (1.1)	0/90 (0)	3/90 (3.3)	0/89 (0)	NA
**Prevalence of vomiting**					
SAC	6/90 (6.7)	3/90 (3.3)	10/90 (11.1)	7/90 (7.9)	2.36 (0.63, 8.83)
**Prevalence of pneumonia^c^**					
SAC	0/90 (0)	3/90 (3.3)	0/90 (0)	3/89 (3.4)	1.01 (0.21, 4.87)

a *Reported blood in stools and/or >5 maximum number of stools in 24-h period.*

b *Reported diarrhoea lasting 7–13 days.*

c *Reported fast, short, rapid breaths due to “chest” or “chest and nose”*.

We were not able to report alpha (1)-acid glycoprotein (AGP) (mg L^−1^) and C-reactive protein (CRP) (mg L^−1^) concentration data, because the COVID-19 pandemic has prevented our transferring samples from Malawi to laboratories for analysis. These data will be reported subsequently.

### Intervention Delivery and Adherence

Over the course of the trial 180 households received a total of 2,754 bags of flour (c. 23,770 kg). Of these, 32 bags (1.16%) were incorrectly distributed, affecting eight households. In the intervention arm, due to a coding error, one household received control flour for the duration of the trial. A further five households in the intervention arm and two households in the control arm received the incorrect flour for some or all of their allocation at a single distribution time point. No data were excluded in this intention-to-treat analysis.

Households reported which flour they had used the previous day on 96% of monitoring instances, and in 98% of these cases they used the trial flour for more than half of meals consumed the previous day (Supplementary Table 4). We did not conduct our planned dose-response analysis, since adherence was so high. There was a large amount of missing data on other proxy measures of adherence, such as quantity of trial flour remaining in the household, and these other measures were not considered in the analysis.

### Adverse Events

At endline, there were more cases of diarrhoea among WRA and SAC in the intervention compared to control arms, although this difference was not statistically significant and reported cases during the intervention period were similar between arms (Table 7; Supplementary Table 5). The incidence was low and was not remarkable for the setting, particularly considering that 104 households (58%) reported getting some or all of their drinking water from an unprotected well, spring or surface water (Supplementary Table 6). The incidence of other AEs was low and did not significantly differ between the control and intervention arms of the trial. There were no serious adverse events reported during the trial.

## Discussion

Our trial has demonstrated that consumption of agronomically-biofortified maize flour led to an improvement in serum Se concentration among women and children in a rural community setting in Malawi. This effect was large and statistically significant. The serum Se concentration data were consistent with the estimates of dietary Se intake.

The intervention had no significant effect on hemoglobin concentrations or anemia prevalence, although the effect on prevalence of anemia among SAC was borderline significant (relative risk 0.73, 95% CI 0.49, 1.09). The causes of anemia are multifactorial (34) and it may be that alleviating Se deficiency in this population is not sufficient to meaningfully affect anemia risk, or the exposure to the intervention was not long enough. However, the increase in serum Se concentrations seen in the intervention arm is likely to be of clinical relevance, with potential implications for thyroid and anti-oxidant functioning. Impaired expression of iodothyronine deiodinase has been reported previously among individuals with serum Se concentrations in the range we observed at baseline (2–4). Hurst et al. (23) reported greater selenoprotein P concentrations among mildly Se-deficient adults following supplementation with 50 or 100 μg day^−1^ of Se, with a steep positive association of plasma Se concentrations with selenoprotein P concentration up to a plasma Se concentration of ~115 μg L^−1^.

The findings are consistent with previous studies that have demonstrated: improved Se status following consumption of supplements, fortified products, and agronomically-biofortified wheat products (22, 23, 35); surveys where human Se status was found to reflect the availability of Se in locally-available foods and soils (15, 16); and repeated cross-sectional data on the Se status of the Finnish population following a national agronomic biofortification policy (21). The present study provides robust evidence that the Se status of individuals living in rural Malawi can be substantially improved through consumption of agronomically-biofortified maize flour. Importantly, this improvement occurred over a relatively short period of time.

The baseline Se status of participants was consistent with the observations of Phiri et al. (1), who reported a low Se concentration in plasma and high likelihood of deficiency among WRA living in the study area. Similar to Phiri et al. (1) and Belay et al. (32), we found serum Se concentrations were greater among WRA than SAC at baseline and endline. The difference between demographic groups may be explained in part by the effect of inflammation, which is more prevalent among SAC than WRA. Inflammation decreases concentrations of circulating Se (3).

Selenium has a relatively narrow range of “optimal” intakes, and excessive intakes leading to toxicity have been reported in other settings (28). In our study, there was no evidence that the intervention risked causing Se toxicity. The greatest serum Se concentration observed in the intervention group was 146 mg L^−1^, well below the concentrations at which mild Se toxicity has been reported to occur of >1,000 μg L^−1^ (28). This is consistent with the dietary data, whereby no WRA had intakes >400 μg day^−1^ the UL of intake. An individual would need to consume an implausible 2.4 kg day^−1^ of the biofortified maize flour to achieve a Se intake of 400 μg, illustrating that biofortification of maize is likely to be a safe approach in Malawi.

Although Se status improved substantially among SAC in the intervention group, 19% had plasma Se concentrations below the threshold for the optimal expression of GPX3 (>84.9 mg L^−1^) at endline. Further research is required to determine the “optimal” application rate of Se for a national agronomic biofortification policy in Malawi to ensure that the dietary requirements of all demographic groups are met, including in areas of Malawi with very low baseline Se intakes, while ensuring minimal risks of excessive intake. Applying Se-containing fertilizers to soils that already have naturally high concentrations of plant-available Se is unlikely to lead to Se concentrations in maize grain that risk excessive intakes (19), however, monitoring of Se intake from all food sources would be appropriate in these areas.

Strengths of the present study include the robust and well-powered trial design, little loss to follow-up, and the high sensitivity of the ICP-MS method. There were several limitations to the study. First, there was no direct measurement of adherence to treatment. There was also a large amount of missing data for some of the proxy measures of adherence, with RAs reporting that households were often reluctant to show them any unused trial flour due to concern that they would receive less flour at the next distribution, and reluctance to hand over empty sacks because the households can reuse these. However, the serum results indicate high adherence. Second, the secondary outcomes relating to morbidity were self-reported and may not accurately reflect the incidence of illnesses, particularly for pneumonia, and these findings should be interpreted with due caution (36). Similarly, pregnancy status was self-reported and this may under-estimate occurrence of pregnancy, especially among those in the first trimester. Third, the study included non-pregnant WRA and SAC only. Fourth, the outcomes were measured at baseline and endline only, after c. 7 weeks' exposure to the intervention (shorter than intended due to insufficient flour), and it cannot be determined whether plasma Se concentrations had plateaued. The steady state of Se status depends on various factors including Se dose, Se chemical form, and interaction with other nutrients. Hurst et al. (23) reported that participants receiving supplements of 50 or 100 μg Se day^−1^ with a selenomethionine content of 60% reached a steady plasma Se concentration after 6 weeks of exposure, while it took up to 10 weeks for those receiving 200 μg Se day^−1^. In maize grain biofortified with Se fertilizer and grown at the same site as the AHHA study maize (37), >90% of the extracted Se was present as selenomethionine with similar speciation profiles of soil and fertilizer-derived Se (38).

Adequate Se status is important for all population groups. Sample size calculations of future studies that include pre-school children (PSC) would need to consider their variable growth rate and smaller meal sizes. In many settings including the current study setting, adult men are frequently away from home meaning they are less likely to consume meals prepared at home, as well as posing challenges for follow up and potential introduction of sampling bias.

The findings of the current study imply that agronomic biofortification can play an important role in alleviating Se deficiency in rural Malawi, and thus contribute toward achieving Sustainable Development Goal 2, to “end all forms of malnutrition” (39). Biofortification is one of the five major focus areas of the National Micronutrient Strategy for Malawi (40), and our study contributes robust evidence that is relevant to the Third Malawi Growth and Development Strategy that calls for “Promoting bio-fortification and fortification of major staple food” (41), and the National Multi-Sector Nutrition Policy 2018–2022 that states the intention to “promote consumption and utilization of…bio-fortified foods.” Application of Se via fertilizer at the required rates are not expected to cause negative environmental effects (37).

The intervention was effective in a population of predominantly subsistence farming households with low socioeconomic status (Supplementary Table 6). Households in these contexts may be considered “hard to reach,” with limited household purchasing power restricting access to diverse diets or foods fortified at processing stage. Only a minority of households in sub-Saharan Africa consume maize flour that is “potentially fortifiable” as most maize is milled at local small-scale mills, and fortification at processing stage is unlikely to reach rural households with low purchasing power (42). Similarly, recent evidence suggests that the majority of Malawian households require greater dietary Se supply, but achieving this through dietary diversification would be prohibitively expensive for most households (43). In contrast, agronomic biofortification appears to be highly cost-effective for addressing Se deficiency, with previous estimates of ~US$ 0.36 year^−1^ per alleviated case of Se deficiency based on input costs (11). Selenium can be incorporated into granular fertilizers during fertilizer production, or included in blends post-production, thus agronomic biofortification with Se could be widely adopted by farmers without any change to their practices. Nationally, 68% of farmers reported applying inorganic fertilizer in 2016–2017 (44). A national agronomic biofortification strategy could operate through the Government of Malawi's Affordable Inputs Programme (the successor to the Farm Input Subsidy Programme) which aims to enable all 4.3 million smallholder farming households in Malawi to access 100 kg of mineral fertilizer (45). Working through this programme may help to reach poorer, rural households who have the greatest baseline risk of Se deficiency (1). Area-specific guidance for Se-containing fertilizers may be warranted to improve cost-efficiency, since their applications is unlikely to deliver benefits in areas of Malawi where agricultural soils already have sufficient concentrations of plant-available Se. An area-specific approach could also minimize any risks of excessive Se intakes.

In conclusion, the present study provides proof of concept that the Se status of women and children in rural Malawi can be improved using an agronomic biofortification approach, and further work is now required to establish the cost-effectiveness and feasibility of scaling this technology, including in other countries in sub-Saharan Africa where Se deficiency is prevalent (32, 46).

## Data Availability Statement

The original contributions presented in the study are publicly available. This data can be found at: https://datacompass.lshtm.ac.uk/ and https://doi.org/10.17037/DATA.00001993. Further inquiries can be directed to the corresponding author.

## Ethics Statement

The study involved human participants and study protocols were reviewed and approved by the University of Malawi College of Medicine Research Ethics Committee (reference P.11/18/2539) and the London School of Hygiene & Tropical Medicine Interventions Research Ethics Committee (reference 16181). Written informed consent to participate in this study was provided by adult participants and the legal guardian of child participants.

## Author Contributions

EJ, AK, JS, AD, EF, GC-P, SL-E, RL, KM, JP, FP, DG, ELA, NL, PN, MB, and EA co-designed the research. EJ, AK, JS, EF, LB, GC-P, HM, ZK, EB, SY, BL, LM, JM-B, KM, JG, PN, MB, and EA conducted the research. EJ, AK, JS, LB, MN, LS, ZK, EF, and EA analyzed data and performed the statistical analysis. EJ drafted the paper. EJ, AK, JS, and EA had primary responsibility for the final content of the paper. All authors read and approved the final manuscript.

## Funding

This work was supported, in whole or in part, by the Bill & Melinda Gates Foundation (INV-009129). Under the grant conditions of the Foundation, a Creative Commons Attribution 4.0 Generic License has already been assigned to the Author Accepted article version that might arise from this submission. The funder had no role in the design, execution, analyses or interpretation of the data. Standard language for the funding acknowledgement is provided by the Gates Foundation in their Open Access Policy.

## Conflict of Interest

The authors declare that the research was conducted in the absence of any commercial or financial relationships that could be construed as a potential conflict of interest.

## Publisher's Note

All claims expressed in this article are solely those of the authors and do not necessarily represent those of their affiliated organizations, or those of the publisher, the editors and the reviewers. Any product that may be evaluated in this article, or claim that may be made by its manufacturer, is not guaranteed or endorsed by the publisher.
