# Supplementary material for: Biofortified Maize Improves Selenium Status of Women and Children in a Rural Community in Malawi: Results of the Addressing Hidden Hunger With Agronomy Randomized Controlled Trial
Source: Front Nutr. 2022 Jan 6;8:788096. doi: 10.3389/fnut.2021.788096 (PMC8770811; doi:10.3389/fnut.2021.788096)
Supplement: Supplementary file 2 [file Table_2.DOCX]

# Supplementary Table 2. Mean (standard deviation) serum selenium concentration at baseline and endline and the mean difference (bias corrected 95% Confidence Interval) at endline between trial arms, among women of reproductive age (WRA) participants by sub-group. Lactation and pregnancy status were self-reported at endline.

|  | **Control** | | **Intervention** | |  |
| --- | --- | --- | --- | --- | --- |
|  | Baseline serum selenium concentration (µg L^–1^) | Endline serum selenium concentration (µg L^–1^) | Baseline serum selenium concentration (µg L^–1^) | Endline serum selenium concentration (µg L^–1^) | Difference in serum selenium concentration (µg L^–1^) |
| ***Age (years)*** |  |  |  |  |  |
| 20 – 30 | 59.8 (18.0)  n=33 | 60.7 (14.1)  n=33 | 57.7 (16.6)  n=24 | 111.5 (13.5)  n=23 | 46.0 (41.0, 51.0)  n=173 |
| 30 – 40 | 63.2 (29.3)  n=39 | 62.1 (14.0)  n=39 | 56.9 (16.0)  n=37 | 104.8 (16.7)  n=39 | 51.0 (44.8, 57.1)  n=173 |
| ≥ 40 | 58.0 (15.3)  n=17 | 59.0 (11.1)  n=16 | 58.6 (19.2)  n=27 | 109.5 (17.9)  n=26 | 51.9 (46.9, 56.9)  n=173 |
| ***Lactation status^1^*** |  |  |  |  |  |
| Not lactating | 63.2 (25.4)  n=65 | 62.6 (14.1)  n=64 | 58.3 (17.2)  n=60 | 107.6 (15.7)  n=60 | 47.9 (44.4, 51.4)  n=170 |
| Lactating | 54.4 (14.7)  n=22 | 56.2 (11.2)  n=22 | 56.1 (17.6)  n=26 | 109.0 (18.3)  n=27 | 52.1 (45.6, 58.6)  n=170 |
| ***Pregnancy status^1^*** |  |  |  |  |  |
| Not pregnant | 61.1 (23.7)  n=85 | 61.0 (13.8)  n=84 | 57.7 (17.4)  n=84 | 108.3 (16.5)  n=85 | 47.9 (44.4, 51.4)  n=170 |
| Pregnant | 56.0 (9.3)  n=2 | 57.8 (0.2)  n=2 | 54.6 (3.6)  n=2 | 94.8 (9.0)  n=2 | 37.7 (25.1, 50.3)  n=170 |

^1^Missing values for control arm baseline (n=2) and endline (n=2), and intervention arm baseline (n=2) and endline (n=1)
