# Supplementary material for: Biofortified Maize Improves Selenium Status of Women and Children in a Rural Community in Malawi: Results of the Addressing Hidden Hunger With Agronomy Randomized Controlled Trial
Source: Front Nutr. 2022 Jan 6;8:788096. doi: 10.3389/fnut.2021.788096 (PMC8770811; doi:10.3389/fnut.2021.788096)
Supplement: Supplementary file 3 [file Table_3.DOCX]

# Supplementary Table 3. Mean (standard deviation) dietary selenium intake at endline and the mean difference (bias corrected 95% Confidence Interval, calculated using the multilevel mixed-effects linear regression) between trial arms, among women of reproductive age (WRA) participants by sub-group. Lactation and pregnancy status were self-reported at endline.

|  | **Control** | **Intervention** |  |
| --- | --- | --- | --- |
|  | Endline intake (µg day^–1^) | Endline intake (µg day^–1^) | Difference in intake (µg day^–1^) |
| ***Age (years)*** |  |  |  |
| 20 – 30 | 28.9 (29.6)  n=32 | 84.7 (46.9)  n=24 | 51.6 (31.8, 71.5)  n=56 |
| 30 – 40 | 29.4 (38.8)  n=39 | 80.6 (37.7)  n=39 | 65.0 (48.2, 81.8)  n=78 |
| ≥ 40 | 21.0 (23.5)  n=17 | 55.5 (9.6)  n=26 | 55.8 (32.6, 79.0)  n=43 |
| ***Lactation status*** |  |  |  |
| Not lactating | 25.6 (28.4)  n=66 | 82.3 (37.3)  n=62 | 54.9 (41.9, 67.9)  n=128 |
| Lactating | 33.7 (44.0)  n=22 | 97.6 (61.1)  n=27 | 62.7 (41.7, 83.6)  n=49 |
| ***Pregnancy status*** |  |  |  |
| Not pregnant | 28.0 (33.1)  n=86 | 87.6 (46.3)  n=87 | 58.0 (46.7, 69.3)  n=173 |
| Pregnant | 10.5 (10.0)  n=2 | 55.5 (9.6)  n=2 | 42.6 (-32.5, 117.6)  n=4 |
