# Supplementary material for: Biofortified Maize Improves Selenium Status of Women and Children in a Rural Community in Malawi: Results of the Addressing Hidden Hunger With Agronomy Randomized Controlled Trial
Source: Front Nutr. 2022 Jan 6;8:788096. doi: 10.3389/fnut.2021.788096 (PMC8770811; doi:10.3389/fnut.2021.788096)
Supplement: Supplementary file 4 [file Table_4.DOCX]

# Supplementary Table 4. Adherence to treatment on days before monitoring visits and the endline survey among households participating in the AHHA trial. Households reported the number of meals that they ate the previous day, and the number of meals in which trial flour was used.

|  | **Monitoring 1** | **Monitoring 2** | **Monitoring 3** | **Endline** |
| --- | --- | --- | --- | --- |
| ***Meals eaten, n (%)*** |  | | | |
| 0 | 2 (1.1) | 0 | 0 | 0 |
| 1 | 2 (1.1) | 1 (0.6) | 1 (0.6) | 1 (0.6) |
| 2 | 43 (24.4) | 42 (24.7) | 41 (23.3) | 23 (12.9) |
| 3 | 129 (73.3) | 127 (74.7) | 134 (76.1) | 155 (86.6) |
| Total households | 176 | 170 | 176 | 179 |
|  |  |  |  |  |
| ***Proportion of meals using trial flour, n (%)*** |  | | | |
| 0 | 3 (1.7) |  |  | 1 (0.6) |
| 33% | 5 (2.9) | 1 (0.6) |  |  |
| 50% | 3 (1.7) |  |  |  |
| 67% | 16 (9.2) | 6 (3.5) | 2 (1.1) | 15 (8.4) |
| 100% | 147 (84.5) | 163 (95.9) | 174 (98.9) | 163 (91.1) |
| Total households | 174 | 170 | 176 | 179 |
