# Supplementary material for: Biofortified Maize Improves Selenium Status of Women and Children in a Rural Community in Malawi: Results of the Addressing Hidden Hunger With Agronomy Randomized Controlled Trial
Source: Front Nutr. 2022 Jan 6;8:788096. doi: 10.3389/fnut.2021.788096 (PMC8770811; doi:10.3389/fnut.2021.788096)
Supplement: Supplementary file 5 [file Table_5.DOCX]

# Supplementary Table 5. Experience of Adverse Events during monitoring visits among women of reproductive age (WRA) and school-aged children (SAC) participating in the AHHA trial.

|  | **Code** | **Description** | **Control, n** | **Intervention, n** |
| --- | --- | --- | --- | --- |
| ***WRA*** |  |  |  |  |
| Diarrhoea | 1 | Yes | 11 | 15 |
|  | 2 | No | 425 | 421 |
|  | 3 | Not sure/would rather not say | 3 | 1 |
| Fever | 1 | No fever | 426 | 417 |
|  | 2 | Fever for 1–2 days | 8 | 12 |
|  | 3 | Fever for 3–4 days | 2 | 7 |
|  | 4 | Fever for 5+ days | 0 | 0 |
|  | 5 | Not sure/would rather not say | 3 | 1 |
| ***SAC*** |  |  |  |  |
| Diarrhoea | 1 | Yes | 12 | 8 |
|  | 2 | No | 422 | 427 |
|  | 3 | Not sure/would rather not say | 5 | 2 |
| Fever | 1 | No fever | 400 | 396 |
|  | 2 | Fever for 1–2 days | 20 | 23 |
|  | 3 | Fever for 3–4 days | 12 | 12 |
|  | 4 | Fever for 5+ days | 4 | 3 |
|  | 5 | Not sure/would rather not say | 4 | 3 |
| Anorexia | 1 | No anorexia | 423 | 422 |
|  | 2 | Anorexia for 1–2 days | 8 | 10 |
|  | 3 | Anorexia for 3–4 days | 2 | 2 |
|  | 4 | Anorexia for 5+ days | 2 | 1 |
|  | 5 | Not sure/would rather not say | 4 | 2 |
| Vomiting | 1 | No vomiting | 424 | 420 |
|  | 2 | Vomiting for 1–2 days | 10 | 11 |
|  | 3 | Vomiting for 3–4 days | 1 | 4 |
|  | 4 | Vomiting for 5+ days | 0 | 0 |
|  | 5 | Not sure/would rather not say | 4 | 2 |
| Cough | 1 | Yes | 79 | 81 |
|  | 2 | No | 356 | 354 |
|  | 3 | Not sure/would rather not say | 4 | 2 |
| Breathing difficulty | 1 | Yes | 8 | 10 |
|  | 2 | No | 427 | 425 |
|  | 3 | Not sure/would rather not say | 4 | 2 |
