# Supplementary material for: Biofortified Maize Improves Selenium Status of Women and Children in a Rural Community in Malawi: Results of the Addressing Hidden Hunger With Agronomy Randomized Controlled Trial
Source: Front Nutr. 2022 Jan 6;8:788096. doi: 10.3389/fnut.2021.788096 (PMC8770811; doi:10.3389/fnut.2021.788096)
Supplement: Supplementary file 6 [file Table_6.DOCX]

# Supplementary Table 6. Socioeconomic status of households participating in the AHHA trial

|  | **Control (n=90)** | **Intervention (n=90)** |
| --- | --- | --- |
|  |  |  |
| Total number of eligible adult women in household, mean (SD) | 1.1 (0.32) | 1.0 (0.11) |
| Total number of eligible school age children in household, mean (SD) | 1.5 (0.69) | 1.6 (0.61) |
| Highest level of education completed by household head, n (%)  No formal education  Primary school  Secondary school  Higher | 3 (3.3)  70 (77.8)  16 (17.8)  1 (1.1) | 5 (5.6)  70 (77.8)  12 (13.3)  3 (3.3) |
| Highest level of education completed by adult woman participant, n (%)  No formal education  Primary school  Secondary school  Higher | 8 (8.9)  71 (78.9)  11 (12.2)  0 (0.0) | 12 (13.3)  67 (74.4)  10 (11.1)  1 (1.1) |
| Primary income source  Subsistence farming  Tobacco  Subsistence farming and tobacco  Subsistence farming, tobacco and other  Subsistence farming & paid employment  Subsistence farming and other  Paid employment  Other | 40 (44.4)  19 (21.1)  20 (22.2)  0 (0.0)  2 (2.2)  1 (1.1)  3 (3.3)  5 (5.6) | 30 (33.3)  24 (26.7)  18 (20.0)  1 (1.1)  1 (1.1)  2 (2.2)  4 (4.4)  10 (11.1) |
| Does the household own land?  No  Yes | 10 (11.1)  80 (88.9) | 9 (10.0)  81 (90.0) |
| Area land owned (if yes)  <0.5 hectares  0.5-2 hectares  2-5 hectares  >5 hectares  Missing | 8 (10.0)  50 (62.5)  14 (17.5)  8 (10.0)  10 (11.1) | 14 (17.3)  40 (49.4)  21 (25.9)  6 (7.4)  9 (10.0) |
| Does the household own their house?  No  Yes | 7 (7.8)  83 (92.2) | 11 (12.2)  79 (87.8) |
| Water source  Tube well or borehole  Protected well  Unprotected well  Unprotected spring  Surface water  Other | 45 (50.0)  0  17 (18.9)  8 (8.9)  20 (22.2)  0 | 37 (41.1)  3 (3.3)  23 (25.6)  4 (4.4)  21 (23.3)  2 (2.2) |
| Toilet facilities  Pit latrine with slab  Pit latrine without slab/open pit  No facility/bush/field  Pit latrine without slab/open pit - shared  Other | 17 (18.9)  35 (38.9)  5 (5.6)  21 (23.3)  12 (13.3) | 17 (18.9)  35 (38.9)  9 (10.0)  18 (20.0)  11 (12.2) |
| Cooking fuel  Charcoal  Wood  Other | 10 (11.1)  79 (87.8)  1 (1.1) | 15 (16.7)  75 (83.3)  0 (0.0) |
| Floor material  Earth/sand  Dung  Cement | 81 (90.0)  3 (3.3)  6 (6.7) | 75 (83.3)  0 (0.0)  15 (16.7) |
| Roof material  No roof  Thatch  Metal | 2 (2.2)  69 (76.7)  19 (21.1) | 0 (0.0)  64 (71.1)  26 (28.9) |
| Wall material  Uncovered adobe  Covered adobe  Bricks  Other | 17 (18.9)  16 (17.8)  53 (58.9)  4 (4.4) | 19 (21.1)  14 (15.6)  51 (56.7)  6 (6.7) |
| Electricity  No  Yes | 89 (98.9)  1 (1.1) | 90 (100.0)  0 (0.0) |
| Radio  No  Yes | 55 (61.1)  35 (38.9) | 54 (60.0)  36 (40.0) |
| Television  No  Yes | 84 (93.3)  6 (6.7) | 83 (92.2)  7 (7.8) |
| Telephone (not mobile)  No  Yes | 89 (98.9)  1 (1.1) | 90 (100.0)  0 (0.0) |
| Computer  No  Yes | 90 (100.0)  - | 90 (100.0)  - |
| Refrigerator  No  Yes | 90 (100.0)  - | 90 (100.0)  - |
| Koloboyi (kerosene lamp)  No  Yes | 88 (97.8)  2 (2.2) | 88 (97.8)  2 (2.2) |
| Paraffin lamp  No  Yes | 89 (98.9)  1 (1.1) | 89 (98.9)  1 (1.1) |
| Lamp/torch  No  Yes | 3 (3.3)  87 (96.7) | 7 (7.8)  83 (92.2) |
| Bed with mattress  No  Yes | 84 (93.3)  6 (6.7) | 74 (82.2)  16 (17.8) |
| Sofa set  No  Yes | 86 (95.6)  4 (4.4) | 79 (87.8)  11 (12.2) |
| Watch  No  Yes | 85 (94.4)  5 (5.6) | 81 (90.0)  9 (10.0) |
| Mobile telephone  No  Yes | 26 (28.9)  64 (71.1) | 28 (31.1)  62 (68.9) |
| Bicycle  No  Yes | 58 (64.4)  32 (35.6) | 47 (52.2)  43 (47.8) |
| Motorbike or scooter  No  Yes | 84 (93.3)  6 (6.7) | 80 (88.9)  10 (11.1) |
| Animal-drawn cart  No  Yes | 89 (98.9)  1 (1.1) | 88 (97.8)  2 (2.2) |
| Car or truck  No  Yes | 89 (98.9)  1 (1.1) | 87 (96.7)  3 (3.3) |
| Boat with a motor  No  Yes | 90 (100.0)  - | 90 (100.0)  - |
| Bank account  No  Yes | 74 (82.2)  16 (17.8) | 70 (77.8)  20 (22.2) |
| Does the household own any livestock?  No  Yes | 28 (31.1)  62 (68.9) | 24 (26.7)  66 (73.3) |
| Milk cows or bulls  None  1 to 4 | 62 (100.0)  0 (0.0) | 64 (97.0)  2 (3.0) |
| Other cattle  None  1 to 4  5 to 9 | 61 (98.4)  1 (1.6)  0 (0.0) | 64 (97.0)  0 (0.0)  2 (3.0) |
| Mules or donkeys  None | 62 (100.0) | 66 (100.0) |
| Goats  None  1 to 4  5 to 9  10+ | 34 (54.8)  21 (33.9)  6 (9.7)  1 (1.6) | 40 (60.6)  16 (24.2)  7 (10.6)  3 (4.5) |
| Pigs  None  1 to 4  5 to 9  10+ | 45 (72.6)  13 (21.0)  4 (6.5)  0 (0.0) | 50 (75.8)  9 (13.6)  6 (9.1)  1 (1.5) |
| Chickens  None  1 to 9  10 to 29  30+ | 7 (11.3)  36 (58.1)  17 (27.4)  2 (3.2) | 5 (7.6)  37 (56.1)  20 (30.3)  4 (6.1) |
| Other poultry  None  1 to 9  10 to 29  30+ | 51 (82.3)  10 (16.1)  0 (0.0)  1 (1.6) | 59 (89.4)  7 (10.6)  0 (0.0)  0 (0.0) |
